# xCT increases tuberculosis susceptibility by regulating antimicrobial function and inflammation

**DOI:** 10.18632/oncotarget.9052

**Published:** 2016-04-27

**Authors:** Yi Cai, Qianting Yang, Mingfeng Liao, Hao Wang, Chi Zhang, Subhalaxmi Nambi, Wenfei Wang, Mingxia Zhang, Junying Wu, Guofang Deng, Qunyi Deng, Haiying Liu, Boping Zhou, Qi Jin, Carl G Feng, Christopher M Sassetti, Fudi Wang, Xinchun Chen

**Affiliations:** ^1^ Guangdong Key Laboratory for Diagnosis and Treatment of Emerging Infectious Diseases, Shenzhen Third People's Hospital, Guangdong Medical College, Shenzhen, China; ^2^ Shenzhen Key Laboratory of Infection and Immunity, Shenzhen University School of Medicine, Shenzhen, China; ^3^ Department of Nutrition, Nutrition Discovery Innovation Center, Institute of Nutrition and Food Safety, School of Public Health, School of Medicine, Collaborative Innovation Center for Diagnosis and Treatment of Infectious Diseases, Beijing Advanced Innovation Center for Food Nutrition and Human Health, Zhejiang University, Hangzhou, China; ^4^ Department of Infectious Diseases and Immunology, Sydney Medical School, the University of Sydney, Sydney, NSW, Australia; ^5^ Department of Microbiology and Physiological Systems, University of Massachusetts Medical School, Worcester, MA, USA; ^6^ Howard Hughes Medical Institute, Chevy Chase, MD, USA; ^7^ Department of Immunology, Bengbu Medical College, Bengbu, China; ^8^ MOH Key Laboratory of Systems Biology of Pathogens, Institute of Pathogen Biology, Chinese Academy of Medical Sciences and Peking Union Medical College, Beijing, China; ^9^ Centre for Tuberculosis, Chinese Academy of Medical Sciences and Peking Union Medical College, Beijing, China

**Keywords:** xCT, tuberculosis, inflammatory, immune, macrophage

## Abstract

The physiological functions of macrophage, which plays a central role in the pathogenesis of tuberculosis, depend on its redox state. System xc-, a cystine-glutamate transporter, which consists of xCT and CD98, influences many ROS-dependent pathways by regulating the production of the antioxidant glutathione. xCT's ability to alter this critical host redox balance by increasing the glutathione synthesis aspect of phagocyte physiology suggested that it might influence tuberculosis pathogenesis. In this study, we found that the xCT expression was increased in peripheral blood monocyte of active tuberculosis. xCT expression in macrophage was induced by Mycobacterium tuberculosis (Mtb) through TLR2/Akt- and p38-dependent signaling pathway. Importantly, xCT deficiency conferred protection against tuberculosis, as xCT knock out mice displayed increased Mtb load and reduced pulmonary pathology in lung compared to wild type mice. xCT disruption enhanced the mycobateriacidal activity of macrophage through increasing the mycothiol oxidation. Importantly, chemical inhibition of xCT with sulfasalazine, a specific xCT inhibitor that is already approved by the FDA for treatment of inflammatory bowel disease, produces similar protective effects *in vivo* and *in vitro*, indicating xCT might be a novel and useful target for host-directed TB treatment strategy.

## INTRODUCTION

Tuberculosis (TB), a chronic bacterial disease caused by *Mycobacterium tuberculosis* (Mtb), remains a major global health problem that claims 1.4 million lives annually. However, only a relatively small proportion of Mtb-infected people develop active disease. The majority of immunocompetent individuals contain the pathogen and remain indefinitely asymptomatic, a status defined as latent tuberculosis infection (LTBI). Recent evidence suggests that inherited differences in the inflammatory profile of TB patients that associates primarily with ethnic variation in host, rather than bacillary genotype, plays a central role in TB disease [1, 2]. Understanding the mechanisms that dictate effective host defense against Mtb infection could suggest new strategies to elicit these protective responses in susceptible individuals.

Macrophage is the first line of host defense against Mtb, and the interaction between the bacterium and macrophage is crucial for determining the outcome of infection [3]. When appropriately activated, macrophages have the capacity to control the replication of intracellular Mtb through multiple mechanisms including the production of reactive oxygen species (ROS) and reactive nitrogen species (RNS), the induction of autophagy and apoptosis/efferocytosis [4]. However, when the macrophage response is insufficient to control bacterial growth, this cell becomes a replication niche for the pathogen facilitating its replication and dissemination [3, 5]. Therefore, the mechanisms by which macrophage integrates activating signals and antimicrobial mechanisms during a protective immune response are central to protective immunity.

The phagocyte antimicrobial response is complex and requires a variety of metabolic adaptations in addition to the simple induction of toxic compounds [6]. The oxidative burst is a clear example of the physiological complexity that accompanies antimicrobial immunity. Upon recognition of an invading microbe, such as Mtb, macrophage assembles an NADPH-oxidase that generates superoxide [7]. This radical is transformed both spontaneously and enzymatically into an array of toxic oxidative species, such as peroxide and hypochlorite. While these compounds are bactericidal, they also have detrimental effects on host cells. ROS can damage important cellular components, particularly essential iron-sulfur proteins [5]. To prevent the damage caused by ROS, macrophages utilize a cytosolic redox buffering system that consists largely of Glutathione (GSH) [8]. As a result, both the antimicrobial and immunoregulatory activities of the macrophage are likely to depend on an appropriate balance between ROS and GSH [5].

The glutamate/cystine antiporter system xc-, which transports cystine into cells in exchange for glutamate at a ratio of 1:1, is composed of a specific light chain, SLC7A11 or xCT and, and a heavy chain, 4F2 or CD98, linked by a disulfide bridge [9, 10]. Once transported into the cell, cystine is rapidly reduced into cysteine, the rate-limiting precursor of GSH. Consequently, induction of system Xc- has been recognized to be essential for oxidative stress resistance in a variety of cell types [11–13]. Considering the important role of xCT in regulating the phagocyte physiology, we hypothesized that xCT might associated with TB pathogenesis by tuning the function of macrophage. In this work, we found that recognition of Mtb infection by innate immune sensors induced xCT expression in macrophages. Disruption of xCT enhanced the antimicrobial activity of macrophages through diminishing GSH production. These effects were also evident in the murine model of TB that *xCT* deficiency simultaneously enhanced bacterial control and reduced the pathological neutrophil accumulation. Chemical modulation of xCT with SASP, an FDA-approved drug for inflammatory bowel disease (IBD), produces similar protective effects *in vivo* and *in vitro*, indicating xCT might be a novel and feasible target for TB treatment.

## RESULTS

### Increased xCT expression is associated with active TB disease in humans

**Figure 1:**
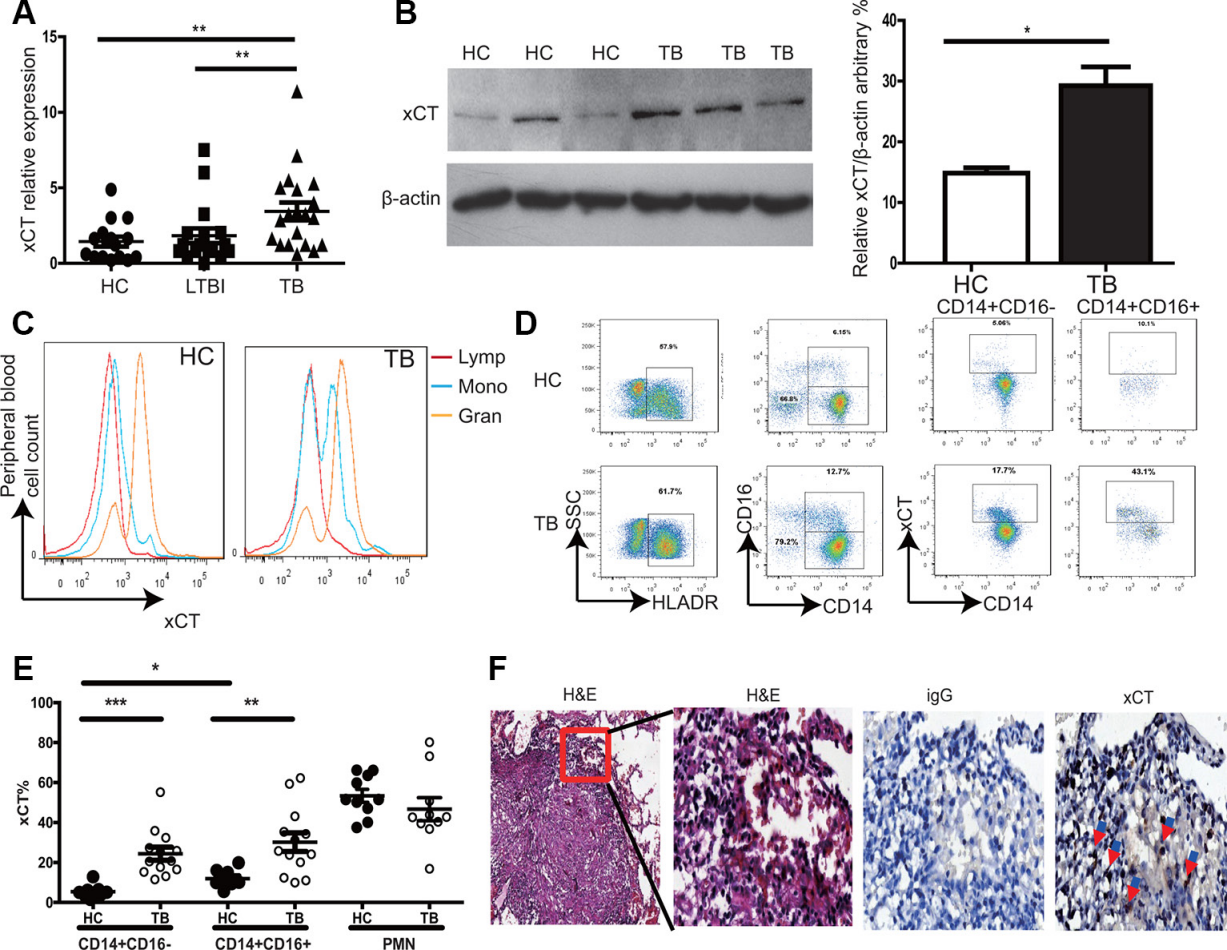
Increased xCT expression in active TB patients (**A**) Expression of *xct* in peripheral blood samples from HC (*n* = 20), LTBI (*n* = 20), and TB (*n* = 20) was determined by qRT-PCR. Relative gene expression was normalized to GADPH. (**B**) xCT protein levels in PBMC from HC (*n* = 3) and TB (*n* = 3) were determined by immunoblotting. Bar graphs showing semi-quantification of xCT level relative to β-actin level, respectively. (**C–E**) Peripheral blood was stained with anti-CD3, CD14, CD16 and xCT antibody and analyzed by flow cytometry. (C) Histogram showed expression of xCT in different cell types from peripheral blood. (D) xCT expression on CD14^+^CD16^−^ and CD14^+^CD16^+^ cells from active TB and HC. (E) The percentage of xCT expressing on CD14^+^CD16^−^, CD14^+^CD16^+^ and PMN cells from active TB (*n* = 10) and HC (*n* = 13). (**F**) Detection of xCT expression in infiltrated immune cells around tuberculous granuloma of lung tissue from patients with active tuberculosis by immunohistochemistry. The brown cells were the xCT positive cells. The one-way ANOVA Newman–Keuls Multiple Comparison Test was used for statistical analyses to compare the differences among multiple groups. Unpaired *t*-test was used to analyze the difference between two groups. *P < 0.05, **P < 0.01, ***P < 0.001.

To investigate whether xCT is associated with the development of active TB disease, we first evaluated *xct* expression in peripheral blood mononuclear cells (PBMCs) from Healthy controls (HC), LTBI, and TB using qRT-PCR. We found that the level of *xct* mRNA was significantly increased in TB compared to HC and LTBI (Figure 1A). In consistence, xCT expression was found significantly higher in PBMCs from TB than those from HC at protein level (Figure 1B). In addition, we found that significantly higher percentages of xCT-expressing CD14^+^ monocytes in peripheral blood from TB patients compared to HC by flow cytometry (Figure 1C–1E). In line with previous report [14], neutrophils constitutively expressed high levels of xCT and lymphocytes did not express this protein (Figure 1C). However, we did not observe any difference in xCT expression in PMN from HC and TB (Figure 1E). Similarly, xCT was highly expressed in tissue macrophage within lung granuloma samples from TB (Figure 1F). Together, these results demonstrated that the expression of xCT on monocytes and macrophages was significantly increased in TB.

### Mtb induces xCT expression via a p38/AKT/TLR2 pathway

**Figure 2:**
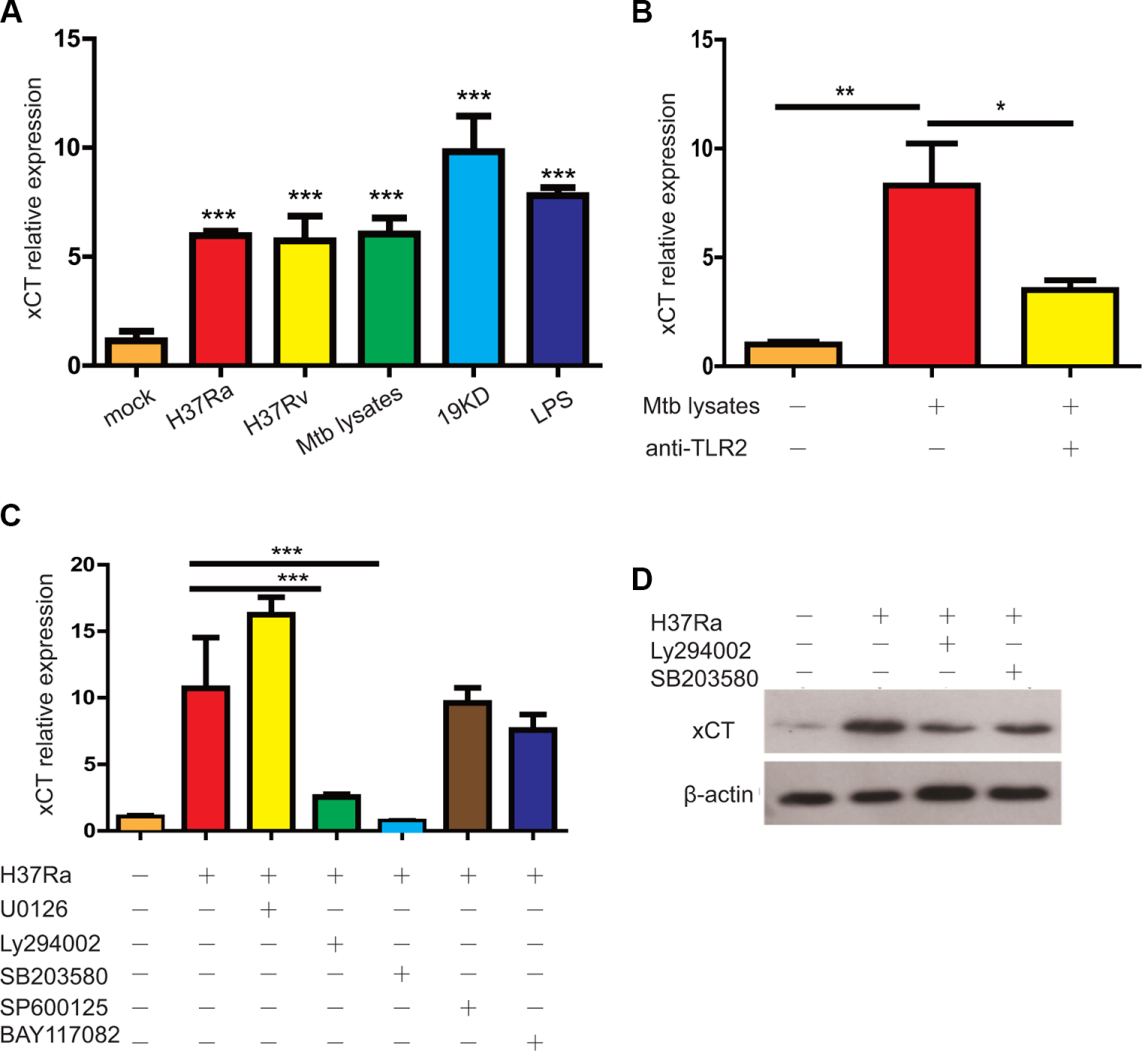
Mtb induced xCT expression in macrophage (**A**) *xct* expression in macrophages infected with H37Ra (MOI = 5), H37Rv (MOI = 5) or stimulated with Mtb lysates 10 μg/ml, 19 kD protein (1 μg/ml), LPS (100 ng/ml). (**B**) *xCT* expression in macrophage treated without or with anti-TLR2 mAb in the absence or presence of Mtb lysate stimulation for 24 hours. (**C**) qPCR detection of *xCT* in U937 macrophage infected with Mtb at MOI of 5 for 24 h in the presence of different kinase inhibitors, SB203580 (10 μM), SP600125 (10 μM), U0126 (10 μM), LY294002 (10 μM), BAY117082 (10 μM). (**D**) Western blot analysis of xCT expression in U937 macrophage infected with Mtb at MOI of 5 for 24 h in the presence of different kinase inhibitors, SB203580 (10 μM), LY294002 (10 μM). (**E**) Western blot of the signaling pathways, p38 and Akt, in U937 macrophage infected with Mtb at MOI of 5 for indicated time. Representative data of 3 experiments were expressed as mean ± SEM *P ≤ 0.05, **P ≤ 0.01, ***P ≤ 0.001.

To investigate the casual relationship between Mtb infection and increased xCT expression in TB, we examined the effect of Mtb infection on *xCT* expression *in vivo* and *in vitro*. As expected, *xCT* expression in the lung tissues was gradually increased in WT mice after Mtb (H37Rv) infection and the difference was statistically significant at 30 days post infection (d30 p.i.) (Supplementary Figure 1). Similar to the *in vivo* data, we found that Mtb infection significantly induced *xct* expression in U937 macrophages (Figure 2A). However, there was no difference of *xCT* expression observed between the virulent H37Rv and avirulent H37Ra strain, suggesting that induction of *xCT* expression was not attributed to the virulence (Figure 2A). In consistence, we found that heat-inactivated Mtb lysate as well as the 19 kD lipoprotein derived from Mtb effectively induced *xCT* expression at least as efficiently as live Mtb (Figure 2A). Since the 19 kD lipoprotein is a well-recognized potent Toll-like receptor 2 (TLR2) agonist [15, 16], the results above suggested that TLR2-signaling could be involved in *xCT* induction. As expected, blocking TLR2-signalling by monoclonal antibody against TLR2 resulted in more than 50% decrease in Mtb-induced *xCT* expression (Figure 2B). On the other hand, the fact that blocking of TLR2 did not completely inhibit Mtb-induced *xCT* expression suggested that other pathways might participate. In line with previous reports, we found that the TLR4 ligand, lipopolysaccharide, also induced *xCT* expression (Figure 2A). Thus, we concluded that the increased xCT expression in TB was a direct effect of Mtb infection, mostly through activating TLR2 pathway.

Next, we further investigated the signaling pathway involved in Mtb-induced *xct* expression by quantification of *xct* expression in H37Ra infected U937 cells with or without PI3K inhibitor (LY294002), ERK inhibitor (U0126), JNK inhibitor (SP600125), p38 inhibitor (SB203580), or NFκB inhibitor (BAY11-7082). As shown in Figure 2C, inhibition of PI3K and p38 pathway, but not JNK and ERK pathway, significantly abolished the effect of Mtb in inducing *xct* expression at mRNA level, which was further confirmed by western blot analysis (Figure 2D).

### Genetic disruption of *xCT* renders mice resistant to TB with decreased lung bacterial burden and reduced pulmonary pathology

**Figure 3:**
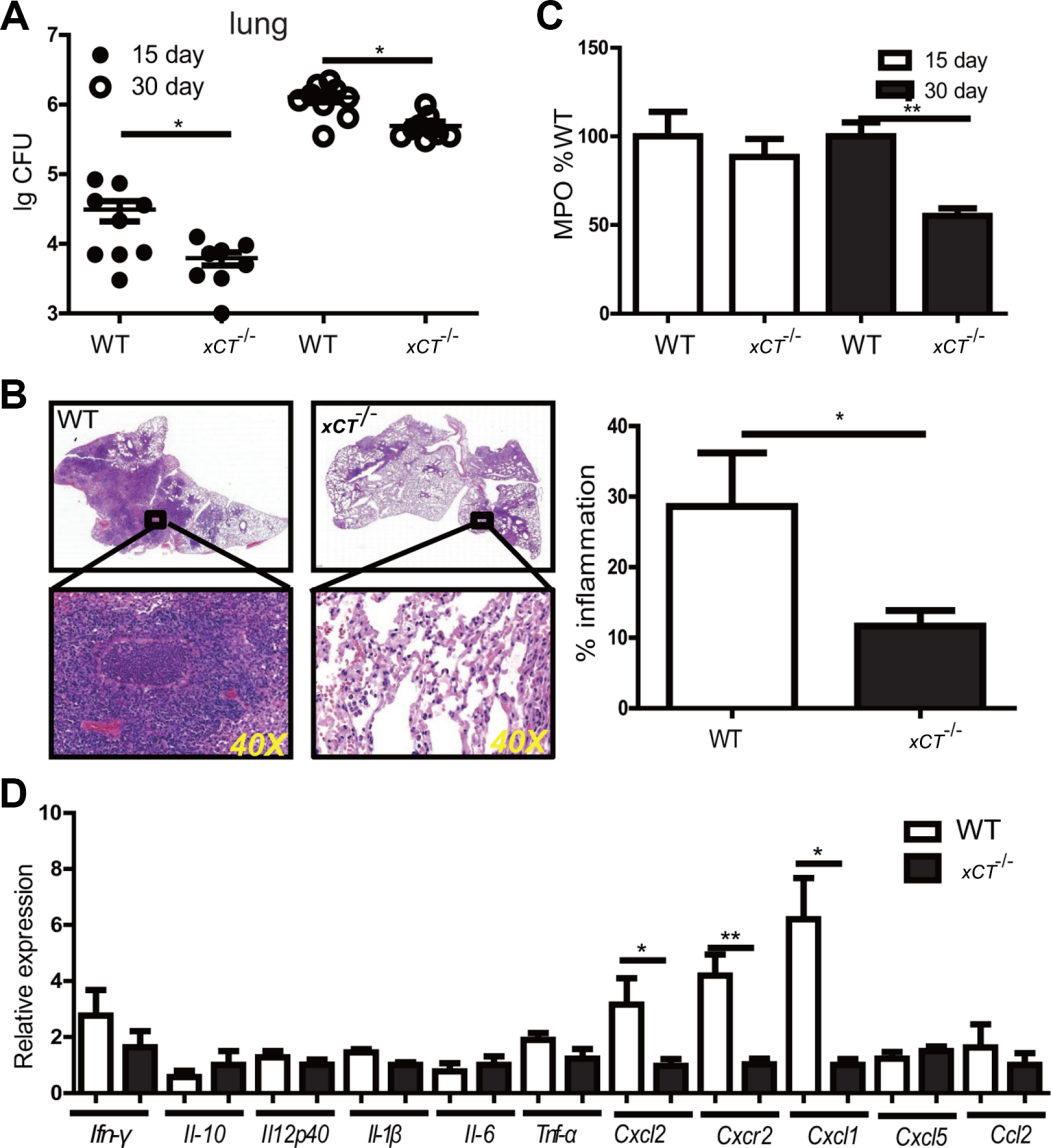
Disruption of xCT renders mice resistant to TB (**A**) Bacterial burden in the lungs of *xCT*^−/−^ and WT (*n* = 4) mice assessed 15–30 d after infection. (**B**) Histopathology of lungs from WT and *xCT* ^−/−^ mice infected with Mtb, stained with hematoxylin and eosin. Outlined areas in main images are enlarged in insets. The lung lopes of HE staining were photographed in digital form using NanoZoomer Digital Pathology System (Hamamatsu Photonics). The percentage of inflammation was determined as the ratio of inflammation area within the whole section of lung tissue area using Nanozoomer 2.0 software (Hamamatsu Photonics). Bar graphs show percentage of inflammation foci in lung from WT and *xCT* ^−/−^ mice. (**C**) The concentration of MPO in the lung homogenate from WT and *xCT*^−/−^ 15 and 30 d after infection. (**D**) The relative expressions of cytokine and chemokine were determined using qRT-PCR in lung homogenates from WT and *xCT* ^−/−^ mice at 30 days after H37Rv infection. Representative data of 2 experiments were expressed as mean ± SEM *P ≤ 0.05, **P ≤ 0.01.

To determine the potential role of xCT in the pathogenesis of TB, we compared outcomes of Mtb infection between wild type (WT) and *xCT* Knock out mice *(xCT*^−/−) [9].^ Compared to the WT mice, *xCT*^−/−^ mice had a significant reduction in bacteria burden in the lung at d15 and d30 p.i. (Figure 3A), indicating that disruption of *xCT* enhanced bacterial clearance. Additionally, the pathology was apparently reduced in the lung of *xCT*^−/−^ mice at d30 p.i., compared with WT mice (Figure 3B). Notably, WT mice developed large and many diffuse lesions that involved 30–40% of the parenchyma, while the *xCT*^−/−^ mice developed small granulomatous lesions occupying about 10% of the lung parenchyma (Figure 3B). Histological examination revealed increased polymorphonuclear neutrophilic infiltration in the lung of the WT mice compared to the *xCT*^−/−^ animals at d30 p.i. In consistence, the concentration of myeloperoxidase (MPO), a surrogate marker of PMN and inflammatory macrophages, was significantly higher in the lung tissue homogenates prepared from the WT mice than those from the *xCT*^−/−^mice at d30 p.i. (Figure 3C). Since chemokines and cytokines are critical to regulate inflammatory and immune responses, we therefore determined the expression of chemokines and cytokines in lung homogenates at d30 p.i. In line with the reduced neutrophils infiltration in the lung of *xCT*^−/−^ mice, the expression of ^*C*^xcl2, Cxcl^1^ and *Cxcr2* were significantly reduced in lung homogenates obtained from *xCT*^−/−^ mice, compared to WT mice (Figure 3D). On the other hand, the expressions of cytokines (*Ifn-γ, Tnf-α, Il-1β, Il-6, Il-10 and Il-12*) and other cytokines (*Cxcl5* and *Ccl2)* were not different between *xCT*^−/−^ and WT mice (Figure 3D). In corroboration with our clinical observation (Figure 1), these data indicated that xCT increases hosts susceptivity to TB.

**Figure 4:**
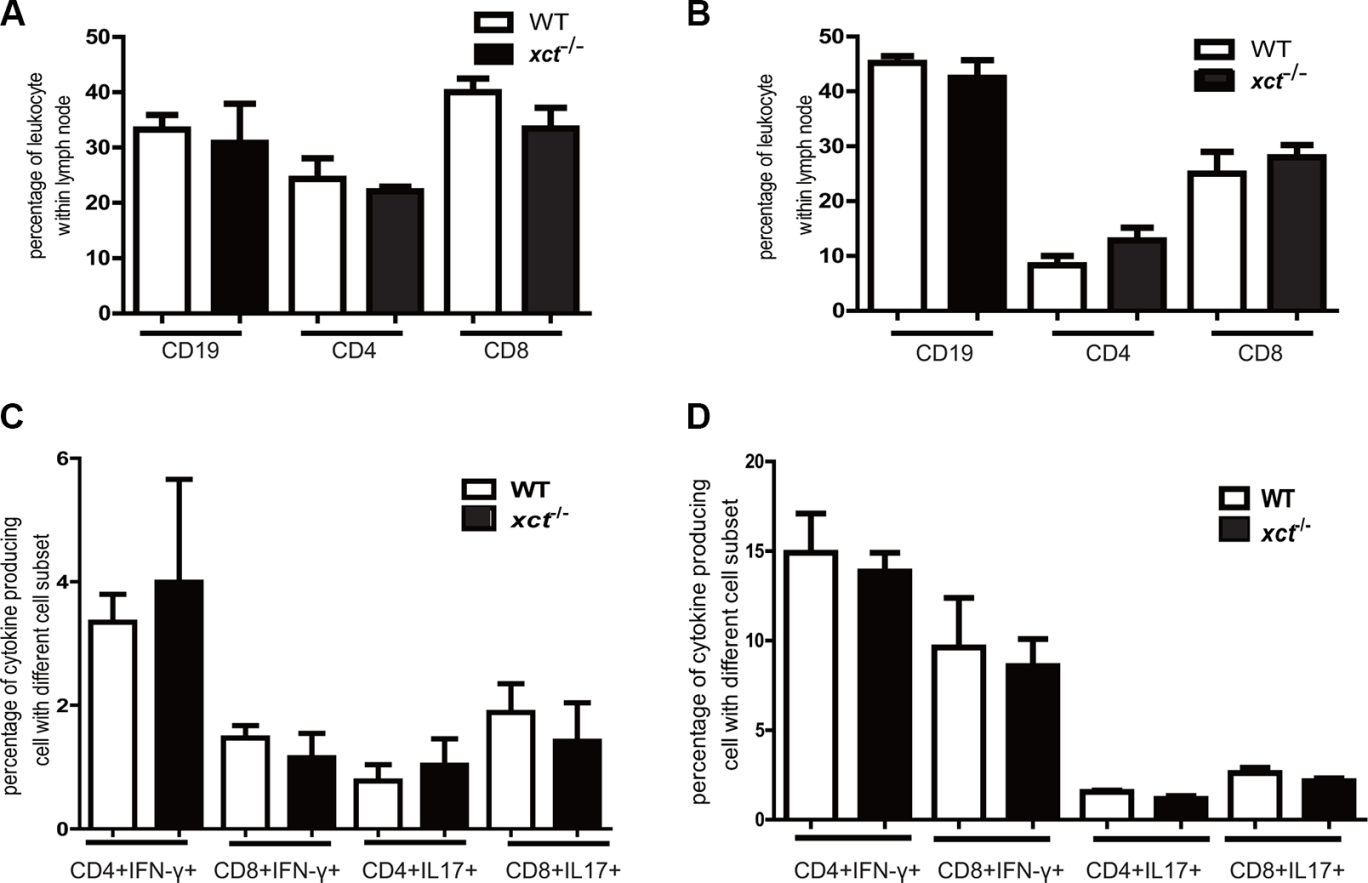
Disruption of xCT does not affect the adaptive immune response in mice infected with Mtb At 30 d p.i., *xCT*^−/−^ (*n* = 4) and WT (*n* = 4) mice were sacrificed, the mediastinal lymph node and spleen were collected from *xCT*^−/−^ and WT mice. Single cell suspension was prepared and subjected for cell surface staining instantly, or cultured in the presence of PMA/ionomycin or Mtb-lysates for intracellular cytokines staining. The percentages of different cell subsets were determined by flow cytometry. (**A**) The percentages of CD4^+^, CD8^+^ and CD19^+^ cells within Mediastinal lymph node and (**B**) spleen from *xCT*^−/−^ and WT mice were determined at 30 day post infection. The frequencies of IFN-γ- and IL-17-producing CD4 and CD8 T cells in spleen after short-term *in vitro* restimulation with Mtb lysate (**C**) or with PMA/ionomycin (**D**) were determined by flow cytometry. Representative data of 2 experiments were expressed as mean ± SEM *P ≤ 0.05, **P ≤ 0.01.

Disruption of xCT has been reported to suppress differentiation and functions of dendritic cells [17], which may in turn inhibit Mtb-specific T cell responses. Since T cell responses are critical to protection against TB, we therefore determined if xCT alters adaptive immunity by monitoring cellular responses in the WT and *xCT*^−/−^ mice after Mtb infection. We found that the frequencies of CD4, CD8 and CD19 cells did not differ significantly between the WT and *xCT*^−^/^−^ mice in the mediastinal lymph nodes (MLN) or spleens (Figure 4A–4B). In addition, Mtb antigen–specific CD4 and CD8 T cell responses, as measured by IFN-γ and IL-17 expression, were comparable in spleen from the WT and *xCT*^−^/^−^ mice (Figure 4C). Similarly, there was no difference of IFN-γ- and IL-17- producing T cells in spleen upon stimulation with PMA/ionomycin (Figure 4D). These data demonstrated that the T cell responses, at least those producing IFN-γ and IL-17, were intact in *xCT*^−^/^−^ mice during Mtb infection, indicating that the resistance to TB conferred by xCT disruption is not associated with adaptive immune response.

### Chemical inhibition of xCT by SASP enhances mycobacterial clearance

**Figure 5:**
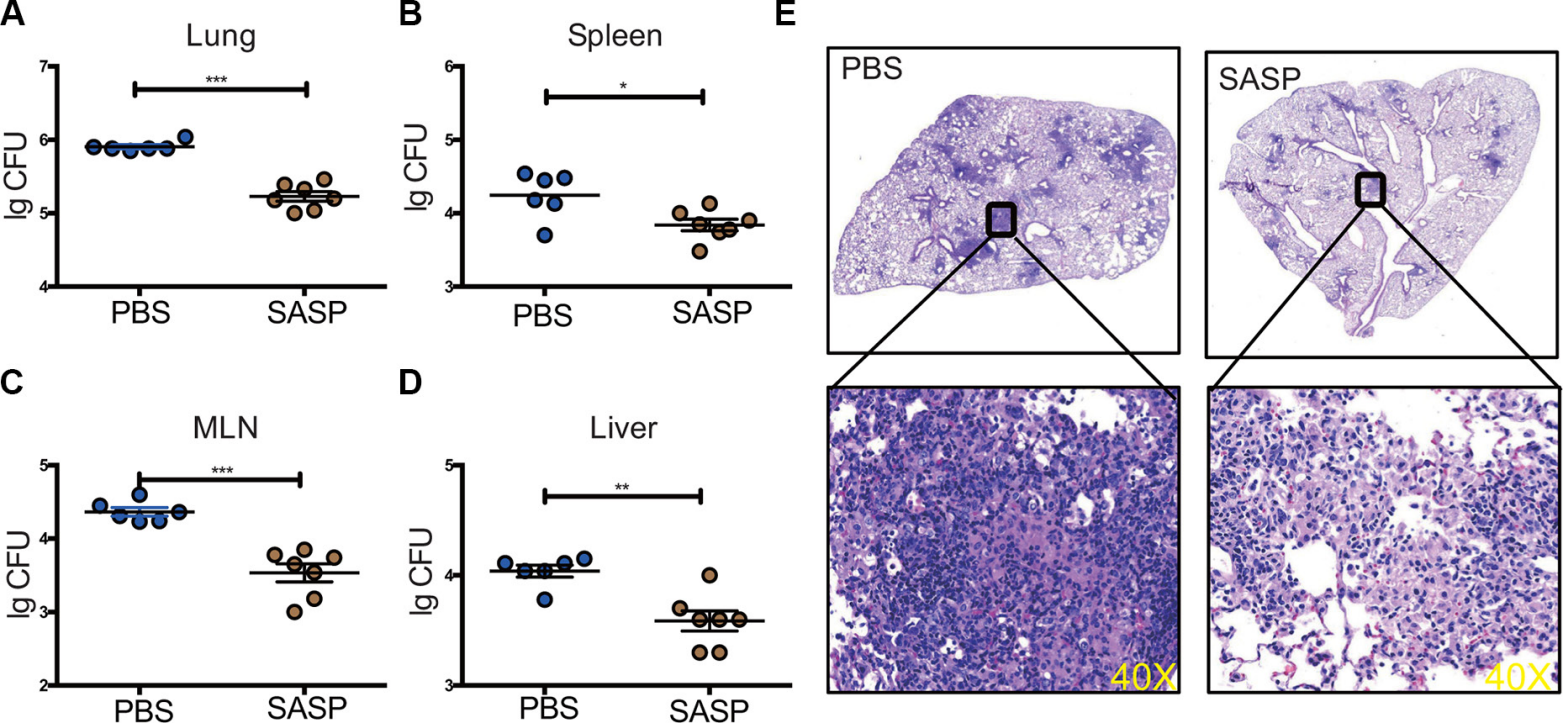
Disruption of xCT by SASP enhanced mycobacteria clearance in mice infected with Mtb Mice infected with H37Rv were gavaged with SASP (20 mg/kg, *n* = 7) or PBS (*n* = 6) at d21 p.i. for another 21 days and then the mice were sacrificed. Bacterial burden in the lungs (**A**), spleens (**B**), MLNs (**C**) and livers (**D**) were assessed by plating of tissues homogenates and presented as Lg CFU. Mean ± SEM reported. *P ≤ 0.05. (**E**) Histopathology of lungs from mice infected with Mtb, stained with hematoxylin and eosin. Outlined areas in main images are enlarged in insets. The lung lopes of HE staining were photographed in digital form using NanoZoomer Digital Pathology System (Hamamatsu Photonics). Magnification, 40×. Experiments repeated at least twice, and mean ± SEM reported. *P ≤ 0.05, **P ≤ 0.01.

Next, we took advantage of SASP, the chemical inhibitor specific for xCT, which had been widely used to disrupt xCT functions previously [18, 19], to determine if the beneficial effects of xCT deficiency could be translated into clinical application against TB. Mtb-infected mice were gavaged with SASP (20 mg/kg) starting on d21 p.i. and sacrificed on d42. Similar to *xCT*^−/−^ mice, mice treated with SASP had significantly reduced bacterial load in the lung, spleen, lymph node and liver compared those treated with PBS (Figure 5A–5D). Histological examination revealed that SASP treatment reduced the lungs pathology with less apparent granulomas formation (Figure 5E). Considering the short-term use of SASP in our murine TB model and the advantage that FDA had already approved SASP for clinical use, these data indicated that modulating xCT is promising for potential clinical application.

### Disruption of xCT enhanced bacterial killing of macrophage through increasing bacterial mycothiol (MSH) oxidation

**Figure 6:**
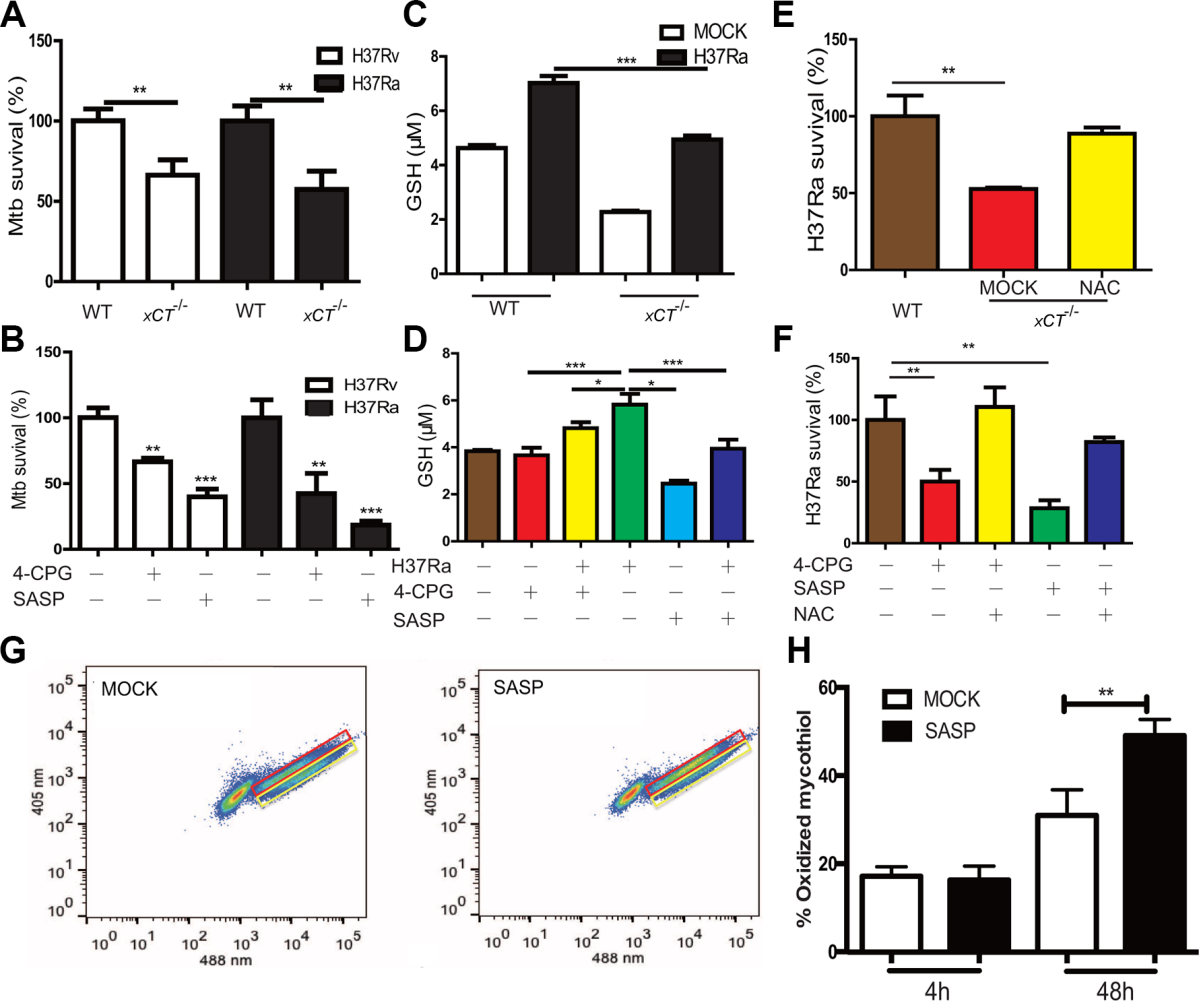
Disruption of xCT enhances macrophages to control mycobacterial infection (**A, C, E**) Peritoneal macrophages from WT and *xCT* ^−/−^ mice were infected with Mtb at MOI of 5 in the absence or presence of NAC (500 μM). (**B, D, F**) U937 macrophages pretreated without or with SASP (200 μM), 4-CPG (100 μM) were infected with H37Ra at MOI of 5 in the absence or presence of NAC (500 μM). (A) CFUs in H37Ra or H37Rv infected macrophage lysates were enumerated at 3d after infection. (B) CFUs in H37Ra or H37Rv infected macrophage lysates were enumerated at 3d after infection. (C) The concentrations of intracellular GSH were evaluated by ELISA at 24 h after infection. (D) The concentrations of intracellular GSH were determined by ELISA at 24 h after infection. (E) CFUs in infected macrophage in the absence or presence of NAC (500 μM) were enumerated at 3 d after infection. (F) CFUs in infected macrophage in the absence or presence of NAC (500 μM) were enumerated at 3 d after infection. H37Ra survival rates were calculated by using mock or WT as a control. (**G, H**) Redox status of intracellular H37Rv population within macrophages. Immortalized C57BL/6 BMDM were infected with H37Rv that was engineered to express Mrx1-roGFP2, and ~30,000 cells were analyzed by flow cytometry by exciting at 405 and 488 nm lasers at a constant emission (510 nm). (G) FACS dot plot of infected macrophages with Mrx1-roGFP2-expressing H37Rv in the absence of SASP and presence of SASP at 48 h p.i. (H) Bar graph represents percentage of oxidized subpopulation of Mtb during different time points post macrophage infection. Values represent the mean ± S.E. of duplicate determinations of experiments repeated twice. Statistical significance was calculated using unpaired student's *t* test. Mean ± SD reported. *P ≤ 0.05, **P ≤ 0.01, ***P ≤ 0.001.

To dissect the mechanisms underlying the beneficial effects of xCT disruption in TB, we investigated whether disruption of xCT directly inhibits bactericidal activity of macrophage. While *xct* deficiency had no effect on the capability of macrophage in uptaking Mtb (Supplementary Figure 2A), *xct* deficiency did significantly enhance the microbicidal activity of macrophage as the number of intracellular Mtb (both H37Ra and H37Rv) was significantly lower in macrophages from *xCT*^−/−^ mice than those from the WT mice at d3 p.i. (Figure 6A). Notably, *xct* deficiency was phenocopied by application of specific xCT inhibitors, (S)-4-carboxyphenylglycine (4-CPG) [20] and SASP. At a concentration (200 nM) without decreasing cell viability (Supplementary Figure 2C–2F), both SASP and 4-CPG significantly enhanced the antimicrobial capacity of macrophage against H37Ra/H37Rv (Figure 6B, Supplementary Figure 2B). The enhancement of antimicrobial in macrophage by SASP was not due to 5-ASA, a metabolic product of SASP, as the addition of SASP in *xCT* deficiency macrophage not further inhibit Mtb growth (data not shown).

Enhancement of autophagy and apoptosis in tumor cells have been reported as critical mechanisms underlying the anti-tumor effect of xCT disruption [18], we therefore evaluated whether apoptosis or autophagy is involved in enhancement of macrophage antimicrobial activity by disruption of xCT, two important mechanisms used by macrophage to kill Mtb [21, 22]. In line with previous reports [21, 22], we found that infection with Mtb significantly induced autophagy and apoptosis of macrophage (Supplementary Figure 2A–2D). However, disruption of xCT had a negligible effect on both events (Supplementary Figure 2A–2D), suggesting that the enhancement of macrophage bactericidal activity by xCT disruption was not due to altered autophagy and apoptosis.

xCT maintains the redox status of the cell by promoting the synthesis of GSH, which scavenges intracellular ROS and NO [23]. We therefore investigated whether the enhancement of antimicrobial activity in xCT-disrupted macrophages was due to altered GSH levels and/or increased ROS and NO activity. As expected, the intracellular GSH levels in macrophages from the *xCT*^−^/^−^ mice were significantly lower than those from the WT mice, either with or without H37Ra infection (Figure 6C). Similarly, disruption of xCT via SASP or 4-CPG significantly decreased GSH level in U937 macrophages upon H37Ra infection (Figure 6D). Moreover, addition of NAC, a precursor of GSH, reversed the enhancement of bactericidal activity by xCT disruption (Figure 6E–6F). Unexpectedly, we did not observe that any effect of xCT disruption on NO and ROS production by macrophages, despite the apparent effect on the production of GSH (Supplementary Figure 3A–3B). In consistence, addition of L-NAME, an inhibitor of NO synthase, did not change bactericidal activity in SASP or 4-CPG treated macrophage (Supplementary Figure 3C). Together, these data demonstrated that xCT disruption enhanced macrophage antimicrobial activity through diminishing GSH production, albeit no apparent effect on the production of NO and ROS was observed at cellular level.

While the data above indicated that disruption of xCT has no effect on ROS/NO production at cellular levels, the enhancement of bactericidal activity was clearly correlated with GSH level, through which a relative increase of ROS and/or ROS/GSH ratio could be expected. We therefore asked whether such a relative increase of ROS and/or ROS/GSH ratio might directly alter the oxidation state of intracellular bacteria [24]. Since the major cytosolic redox buffer of mycobacteria is the cysteine glycoconjugate, MSH, which is different from host GSH; the redox potential of the MSH pool can be specifically determined *in situ* using a ratiometric roGFP reporter fused to the specific MSH reductase Mrx1 [24]. By taking advantage of Mtb that was engineered to express the Mrx1-roGFP reporter, we determined the effect of xCT disruption on the oxidation state of intracellular mycobacteria. We found that disruption of xCT by SASP had no effect on the MSH redox state of H37Rv cultured in Middlebrook 7H9 Broth (Data not shown). In contrast, SASP significantly increased the oxidized MSH pool of H37Rv within infected macrophages at 48 h p.i. (Figure 6G–6H). Since high oxidation renders mycobacteria succumb to death [24], these data indicated that the increase of oxidative stress in intracellular mycobacteria was a critical mechanism for enhancement mycobateriacidal activity in xCT disruption macrophage.

## DISCUSSION

Understanding the key regulatory mechanisms or molecules that dictate host resistance or susceptibility to Mtb infection is important for developing novel strategies for TB control. This is particularly important as MDR- and XDR- Mtb strains that are resistant to conventional anti-TB treatment become more common. Recently, several host molecules have been identified as potential host-directed therapy (HDT) targets [16, 25–27]. In this study, we found for the first time that xCT increases tuberculosis susceptibility by inhibiting antimicrobial function and inflammation and xCT might be a novel target for HDT against TB.

Clinically, increased xCT expression was associated with development of active TB. In mice, disruption of xCT resulted in reduced bacterial load and pathology in the lung. Importantly, we found that disruption of xCT by pharmacological inhibitor, SASP, had similar effects as *xct* genetic deficiency. Therefore, our findings provided an important translational foundation for developing a novel host-direct therapy against TB.

In line with KSHV infection, we found that *xct* deficiency or disruption of xCT by SASP enhanced intracellular mycobacteria killing activity in macrophage through diminishing GSH production. Replenishment of intracellular GSH levels by NAC, a precursor of GSH, abolished the enhanced killing activity of xCT-disrupted macrophages. However, unlike the case of KSHV infection, we found that the NO and ROS levels in xCT-disrupted macrophages upon infection with Mtb were comparable to those in WT macrophages. Nevertheless, the decrease of GSH resulted in altered ROS/GSH ratio, which was mirrored by the oxidation state of the mycobacterial MSH pool, suggesting that these redox buffers are directly or indirectly coupled. Consistently, we found that decrease in GSH levels in macrophage correlated with a more highly oxidized MSH pool in the intracellular mycobacteria. Several studies have found that intracellular bacterial pathogens can link their oxidation state to that of the host cell. For example, host glutathione can activate a critical virulence-associated transcription factor in the cytosol of *L. monocytogenes* [28] and *F. tularensis* acquires GSH from the host cell as a source of cysteine and to maintain its cytosolic redox buffer [29]. However, in the majority of these cases, the precise mechanism linking the redox buffering systems of these two cells remain unclear. In the case of *F. tularensis*, a GSH import system seems to be important. Interestingly, Mtb expresses an analogous dipeptide permease that contributes to the intracellular growth of Mtb [30, 31]. Thus, we hypothesize that host GSH is likely available to the bacterium as an alternative source of reducing potential to its endogenous mycothiol-based buffering system.

The effect of NAC on macrophage mycobacterial killing is complex. We found that NAC abolished the enhancement of macrophage microbicidal activity conferred by xCT-disruption. Similarly, Roca et al. reported that NAC abolished TNF-α induced ROS production and therefore reduced macrophage mycobacterial killing activity [5]. In contrast, enhancing GSH levels by NAC supplementation has also been shown to increase the BCG killing by macrophages or whole blood [32, 33]. Since these latter effects appeared to depend on NO production to form toxic *S*-nitrosoglutathione (GSNO) [32], the disparity the effect of NAC might result from the difference of NO production in these systems. Consistent with this model, both xCT disruption in this study and TNF-α in Roca et al. report had no effect on modulating NO production [5].

With the purpose of translational medicine, we demonstrated that chemical modulation of xCT with SASP, a specific xCT inhibitor that was already approved by the FDA for use in humans, produces similar protective effects of *xct* deficiency *in vivo* and *in vitro*. SASP therapy significantly reduced the bacterial burden in the lung, draining lymph node, spleen, and liver *in vivo*. In consistence with the decreased bacterial burden, the histopathology was also reduced in the lung.

In summary, we demonstrated that host xCT increases host susceptible to TB. Genetic disruption of xCT or chemical modulation of xCT with SASP significantly decreased the bactericidal burden and reduced histopathology in murine TB. By taking advantage of FDA approved use of SASP in human, our findings provided fundamental data for the potential clinical application of xCT as novel target for HDT against TB.

## MATERIALS AND METHODS

### Ethics statement

The Research Ethics Committee of Shenzhen Third People's Hospital approved the collection of peripheral blood exclusively for research purposes with the written informed consent of all participants (No. 2012–007). All mice experimental procedures were performed in accordance with the Regulations for the Administration of Affairs Concerning Experimental Animals approved by the State Council of People's Republic of China. The animal experiments were approved by the Animal Research Ethics Committee of Shenzhen Third People's Hospital (No. 2012–A003).

### Subjects and clinical sample collection

Whole blood samples were collected from healthy controls (HC, *n* = 20), individuals with latent tuberculosis infection (LTBI, *n* = 20), and patients with active tuberculosis (TB, *n* = 20) at Shenzhen Third People's Hospital. Diagnosis of active TB and LTBI was described previously [34]. Briefly, diagnosis of active TB was based on clinical symptoms, chest radiography, and microscopy for acid-fast bacilli (AFB), sputum Mtb culture, and response to anti-TB chemotherapy. A previously established Mtb specific interferon-gamma release assay (IGRA) was used to differentiate individuals with LTBI from healthy controls [35]. Detailed demographic characteristics are listed in Supplementary Table 1. All TB patients were positive for sputum Mtb culture. Whole blood was used for flow cytometry analysis or RT-PCR measurement of xCT expression. For western blot analysis, PBMCs were isolated from whole blood through density gradient centrifugation over Ficoll-Hypaque as described elsewhere [36].

### Culture of cell lines and bacteria

Human leukemic monocyte lymphoma cell line U937 cells (ATCC CRL 1593), were cultured in RPMI1640, supplemented with 10% fatal bovine serum (FBS), antibiotics (50 U/ml penicillin and 50 μg/ml streptomycin), 0.1 mM non-essential amino acids and 1 mM sodium pyruvate in 5% CO_2_ at 37°C. U937 cells were pretreated with 20 ng/ml PMA for 24 h to differentiate them before infection with Mtb. Two mycobacterium strains, H37Ra and H37Rv were grown at 37°C in Middlebrook 7H9 broth (BD) with 0.05% Tween-80 and 10% oleic acid albumin-dextrose-catalase (OADC) enrichment (BD). The inoculum was titrated by plating serial dilutions on Middlebrook 7H11 medium plus 10% OADC.

### Murine TB model

C57BL/6 mice and *xCT*^−/− mice^ (C57BL/6 background) at 8–10 weeks old were used for Mtb infection. *xCT*^−/−^ mice that had been backcrossed at least six times to C57BL/6 mice were provided by professor Shiro Bannai [37] and kept in Dr. Fudi Wang's Lab. Mtb infection was performed with the virulent strain H37Rv. Mice were infected with H37Rv (~150 CFU) using a Glas-Col inhalation exposure system (Glas-Col, USA). At indicated time points, mice were sacrificed and the lungs were collected for bacterial burden enumeration, histopathology, and cytokines/chemokine measurements. Bacterial burdens were evaluated at different time points by homogenizing the lungs in PBS, and plating serial dilutions onto Middlebrook 7H11 agar plates. After 3 weeks, Mtb colonies were counted. For drug treatment, mice were gavage administered SASP (20 mg/kg) or PBS every two days at d21 p.i. Mice were sacrificed at d 42 after infection. The lungs, spleens, lymph node, and livers were collected and CFUs were determined as described above.

### Flow cytometry

Fresh whole blood samples (200 μl each) from HC and TB patients were used for flow cytometry analysis of xCT expression. Briefly, erythrocytes were lysed with lysing solution (BD Biosciences) and then the samples were stained with surface mAbs against CD14 (BD Biosciences), HLA-DR (Immunotech), xCT (Thermo scientific) and CD16 (BD Biosciences). After incubating at room temperature for 15 min, the samples were stained with Alexa Fluor^®^ 647 Goat Anti-Rabbit IgG Antibody (Invitrogen) for additional 15 min at room temperature. At least 0.2 million cells were acquired for analysis using FACSDiva software (BD Biosciences).

Lymph nodes and spleens from the Mtb-infected animals were used for flow cytometry experiments. Single cell suspensions were obtained from spleen and Lymph nodes by grinding the organ through a 70-μm strainer. Erythrocytes were removed with lysing solution (BD Biosciences), and the samples were surface stained with mAbs against CD3, CD19 and CD4 (BD Biosciences). For the intracellular staining of cytokines, spleen cells were stimulated with PMA (10 ng/ml, Sigma-Aldrich), ionomycin (1 m g/ml, Sigma-Aldrich) and brefeldin A (Sigma-Aldrich) for 6 h or with Mtb lysates for 24 h and incubated for the last 6 h with brefeldin A (10 mg/ml). Cells were stained with anti-CD4 and anti-CD3 in HBSS (containing 1% FBS), fixed with 2% formaldehyde, and permeabilized with 0.5% saponin. The cells were subsequently stained with anti-IL-17 (eBioscience) and anti-IFNγ (eBioscience). Cells producing IFNγ and IL-17 were examined with flow cytometry.

### RNA extraction and qRT-PCR

Whole blood samples collected from individuals classified as HC, LTBI and TB using PAXgene Blood RNA tubes (BD Biosciences). Total RNA was extracted using the PAXgene Blood RNA Kit (Qiagen) following the manufacturer's instructions. For cell samples collected from cell culture and lung, total RNA was extracted using QIAamp RNA Mini Kit (Qiagen) following the manufacturer's instructions. The RNA yield and A260/280 ratio were monitored with a NanoDrop ND1000 spectrometer. Purified RNA was reverse transcribed to cDNA using PrimeScript^®^RT reagent Kit (TaKaRa). qPCR was performed using SYBR Green PCR Master Mix (TaKaRa) following the standard protocol. The relative mRNA expression of different genes was calculated by comparison with the housekeeping gene GAPDH using the 2^−ΔΔCt^ method [38], as described previously [34]. The primers used are listed in Supplementary Table 2.

### Isolation of mouse macrophages

Peritoneal macrophages were isolated as described previously [39]. Briefly, C57BL/6 mice and *xCT*^−/−^ mice were injected intraperitoneal with 2 ml of 3% thioglycollate medium (Sigma-Aldrich). After 3 days, cells were harvested by peritoneal lavage with cold PBS and allowed to adhere for 2 h. Non-adherent cells were removed by washing twice with PBS. The remaining adherent cells were cultured in DMEM (10% FBS) and used as primary peritoneal macrophages after at least 24 h. Bone marrow derived macrophage (BMDM) was obtained for WT C57BL/6 mice. Bone marrow cells from the tibia and femur were cultured in DMEM containing 10% FBS and 20 ng/ml recombinant mouse M-CSF. After 5 days, differentiated BMDMs were incubated in 10% FBS DMEM media.

### Mycobacterial infection in macrophages

Peritoneal macrophages and differentiated U937 cells were infected with H37Rv or H37Ra at an MOI of 5. After 4 h incubation, non-internalized bacteria were washed away with PBS and incubation with fresh complete medium. For CFU assays, cells were incubated for 0 and 72 h in fresh complete DMEM. After incubation, cells were lysed with 0.1% SDS and cell lysates were diluted, plated on 7H11 Middlebrook agar plates, and incubated at 37°C in 5% CO_2._ CFU were counted 3–4 weeks later. For gene expression assay, cells were harvested at 24 after infection and subjected to RNA isolation or immunoblotting analysis. In some experiments, cells were pretreated with SASP, 4-CPG (Bioying), NAC (Beyotime), anti-TLR2 (Novus Biologicals), LY294002, BAY117082, U1026, SB203590 or SP600125 (Sellechchem) for 1 h.

### MPO assays

For MPO assays, the lungs were perfused with PBS, then the collected lung lobes were immediately dispersed in PBS supplemented with a protease inhibitor cocktail (Roche) and frozen at −20°C until analysis. Samples from lung homogenates were thawed and appropriately diluted. The concentration of MPO was evaluated using the MPO ELISA kit following the manufacturer's instruction (USCNK).

### Western blotting analysis

The cell pellet was lysed using a western lysis buffer (Beytime) supplemented with a cocktail of protease inhibitors (Sigma-Aldrich). The lysates were quantified and equivalent amounts of proteins were next separated by SDS-PAGE on a 12% polyacrylamide gel and then transferred onto a nitrocellulose membrane. The membranes were sequentially probed with the respective primary antibodies, followed by appropriate HRP-conjugated secondary antibodies (Abcam) and then visualized by exposure to X-ray film. Images were quantified by densitometry using Quantity One, version 4.5 software (Bio-Rad).

### Redox status study

To study the redox state of mycothiolof mycobacteria *ex vivo*, Mtb (H37Rv strain) expressing Mrx1-roGFP2 was grown in Middlebrook 7H9 broth (Difco) supplemented with 10% OADC, 0.2% Glycerol and 0.1% Tween 80. Immortalized C57BL/6 BMDM seeded at 1 × 10^5^ cells per well in 24-well plates treated with or without SASP were infected with Mrx1-roGFP2 expressing H37Rv at an MOI of 10 and incubated for 1 h and 4 h respectively, at 37°C in 5% CO2. Extracellular bacteria were removed by washing twice with PBS. At different time points, infected macrophages were washed with PBS and treated with 10 mM NEM for 5 min at 22°C followed by fixation with 4% PFA for 15 min at 22°C. After washing thrice with PBS, cells were scraped and analyzed using a MACSQuant Flow cytometer. The ratio of emission (510/10 nm) after excitation at 405 and 488 nm was calculated. Data was analyzed using the FlowJo software.

### Immunohistochemistry

Segments of lung tissue from mice or patient with active tuberculosis were fixed in 10% buffered formalin (Sigma-Aldrich) and embedded in paraffin. Histologic sections were stained with H&E for evaluation of pathology. Briefly, after hematoxylin and eosin staining, the whole microscope slide images were photographed in digital form using NanoZoomer Digital Pathology System (Hamamatsu Photonics). The percentage of inflammation was determined as the ratio of inflammation area within the whole section of lung tissue area using Nanozoomer 2.0 software (Hamamatsu Photonics) [40]. For immunohistochemistry, five micron-thick paraffin sections were collected on charged slides, deparaffinized, and re-hydrated in water. After antigen retrieval in pH 6.0 citrate buffer for 5 min at 125°C in a pressure cooker, sections were incubated with primary antibody against xCT for 2 h room temperature. Immunoreactions were revealed by a biotin-free dextran-chain detection system (Envision, DakoCytomation, Glostrup, Denmark) and were developed using 3′,3′-diaminobenzidine as the chromogen.

### GSH assays

Intracellular GSH concentration was determined by using a GSH-Glo™ Glutathione assay kit (Promega, USA) following the manufacturer's instructions.

### Statistical analysis

The one-way analysis of variance/Newman-Keuls multiple comparison test was used for statistical analyses to compare the differences among multiple groups. The unpaired *t* test was used to analyze the difference between two groups. We used GraphPad Prism software (version 5.0) for all the statistical analysis. Two-tailed statistical tests were conducted with a significance level of 0.05.

## SUPPLEMENTARY MATERIALS FIGURES AND TABLES


