# Supplementary material for: xCT increases tuberculosis susceptibility by regulating antimicrobial function and inflammation
Source: Oncotarget. 2016 Apr 27;7(21):31001–13. doi: 10.18632/oncotarget.9052 (PMC5058734; doi:10.18632/oncotarget.9052)
Supplement: Supplementary file 1 [file oncotarget-07-31001-s001.pdf]

xCT increases tuberculosis susceptibility by regulating antimicrobial function and inflammation

Supplementary Materials

SUPPLEMENTARY METHODS

qRT-PCR

Macrophages were infected with H37Ra or H37Rv at a moi of 5, and cells were harvested at indicated time after infection. Total RNA was extracted using QIAamp RNA Mini Kit (Qiagen) following the manufacturer's instructions. The RNA yield and A260/280 ratio were monitored with a NanoDrop ND1000 spectrometer. Purified RNA was reverse transcribed to cDNA using PrimeScript®RT reagent Kit (TaKaRa). qPCR was performed using SYBR™ Green PCR Master Mix (TaKaRa) following the standard protocol. The relative mRNA expression of different genes was calculated by comparison with the housekeeping gene GAPDH using the $2^{-\Delta\Delta Ct}$ method. The primers used are listed in Table S2.

ROS analysis

To measure the intracellular ROS levels, cells pretreated with or without SASP were infected with H37Ra for 48 h and incubated in the dark with 5 μM oxidation-sensitive fluorescentprobe 2′7-dichlorofluorescein-diacetate (DCFH-DA, Beyotime) for 30 min. The strained cells were collected and resuspended in PBS, then subjected to flow cytometry analysis.

Determination of NO production

U937 macrophages pretreated with or without SASP were infected with H37Ra or H37Rv for 24 h, and culture supernatants were collected. The NO levels were measured in the supernatants using the Griess reaction (Beyotime).

Apoptosis analysis

U937 macrophages pretreated with or without SASP and peritoneal macrophages from WT and *xCT*$^{-/-}$ mice were infected with H37Ra for 24 h. Cells were harvested and resuspended in PBS. The cell apoptosis was determined by flow cytometry using Annexin-V-FITC Apoptosis Detection Kit (BD Biosciences) according to the manufacturer's instructions.

Cell viability

Cellular viability was determined using MTT (Sigma-Aldrich) colorimetric assay. U937 macrophages stimulated with PMA were plated in 96-well plates and left overnight to adhere. Cells were then treated at indicated concentrations of drug or H37Ra. At 24 or 72 h, media was replaced with 50 μL of RPMI media (Life Technologies) containing 0.5 mg/ml MTT. The cells were then incubated for 4 h at which point 150 μl DMSO was added to each well. After another 15 min incubation, absorbance was measured at 540 nm using a spectrophotometer.

Western blot

The cell pellet was lysed using a western lysis buffer (Beytime) supplemented with a cocktail of protease inhibitors (Sigma-Aldrich). The lysates were quantified and equivalent amounts of proteins were next separated by SDS-PAGE on a 12% polyacrylamide gel and then transferred onto a nitrocellulose membrane. The membranes were sequentially probed with LC-3β antibody (Sigma-Aldrich), followed by appropriate HRP-conjugated secondary antibodies (Abcam) and then visualized by exposure to X-ray film.

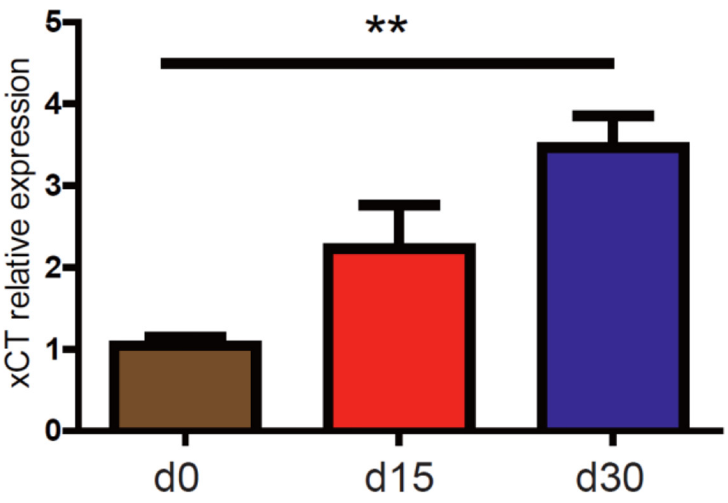

Supplementary Figure S1: Expression of *xCT* during murine TB. qRT-PCR of *xCT* mRNA in the lungs of Mtb infected C57BL/6 mice. Data are presented as the mean ± SEM from two independent experiments. Relative gene expression was normalized to GADPH. Mean ± SEM reported. $^{**}P \leq 0.01$.

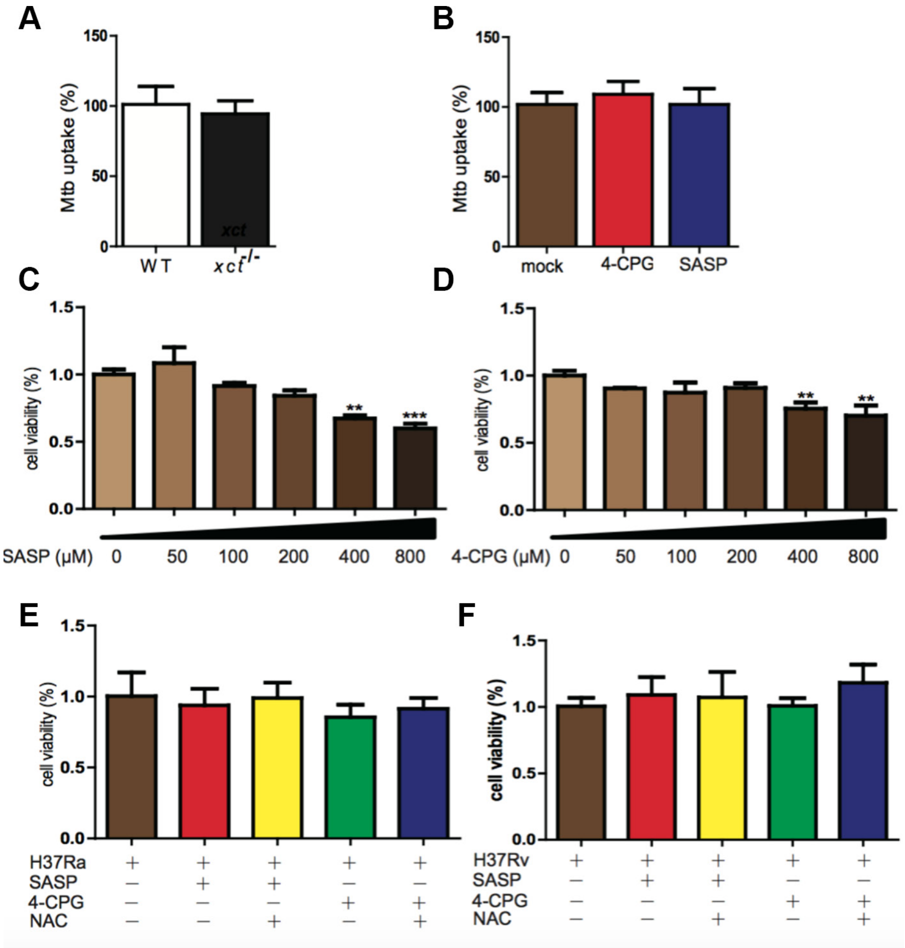

Supplementary Figure S2: Disruption of xCT does not affect Mtb uptake and cell viability. (**A**) Peritoneal macrophages from WT and *xCT*$^{-/-}$ mice were infected with H37Ra at MOI of 5 and CFUs in infected macrophage lysates were enumerated at 4 h after infection. (**B**) U937 macrophages pretreated without or with SASP (200 μM), 4-CPG (100 μM) were infected with H37Ra at MOI of 5 and CFUs in infected macrophage lysates were enumerated at 4 h after infection. (**C**) The effect of different concentrations of SASP on U937 cell viability at 24 h after treatment. (**D**) The effect of different concentrations of 4-CPG on U937 cell viability at 24 h after treatment. The effect of different drug on H37Ra (**E**) or H37Rv (**F**) infected U937 cell viability at 72 h after treatment. Mean ± SEM. $^{**}P \leq 0.01$, $^{***}P \leq 0.001$.

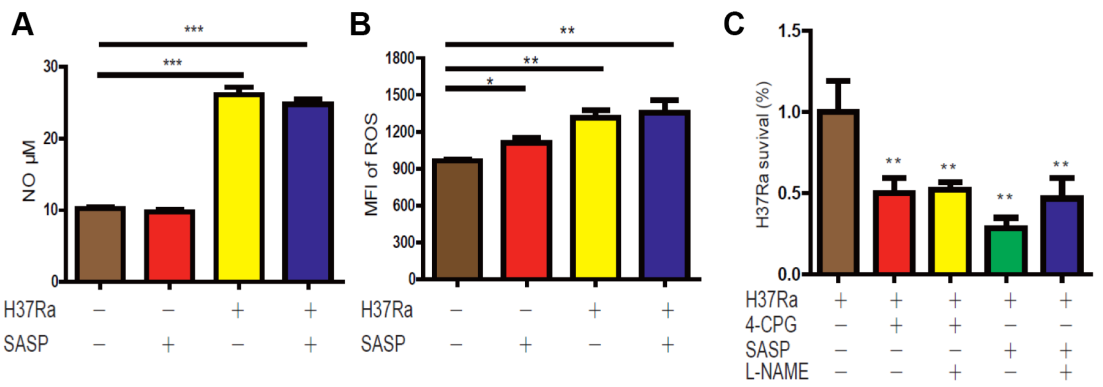

Supplementary Figure S3: Disruption of xCT did not affected ROS and NO production during Mtb infection. U937 macrophages pretreated with or without SASP were infected with H37Ra. After 24 h, supernatants were harvest for NO quantification (**A**) and cells were harvest for determine the ROS levels (**B**); (**C**) U937 macrophages pretreated with or without SASP, 4-CPG were infected with H37Ra at MOI of 5 in the absence or presence of L-NAME (500 μM). CFUs in infected macrophage were enumerated at 3 d after infection. H37Ra survival rates were calculated by using mock as a control. Mean ± SEM. *$P \leq 0.05$ **$P \leq 0.01$, ***$P \leq 0.001$.

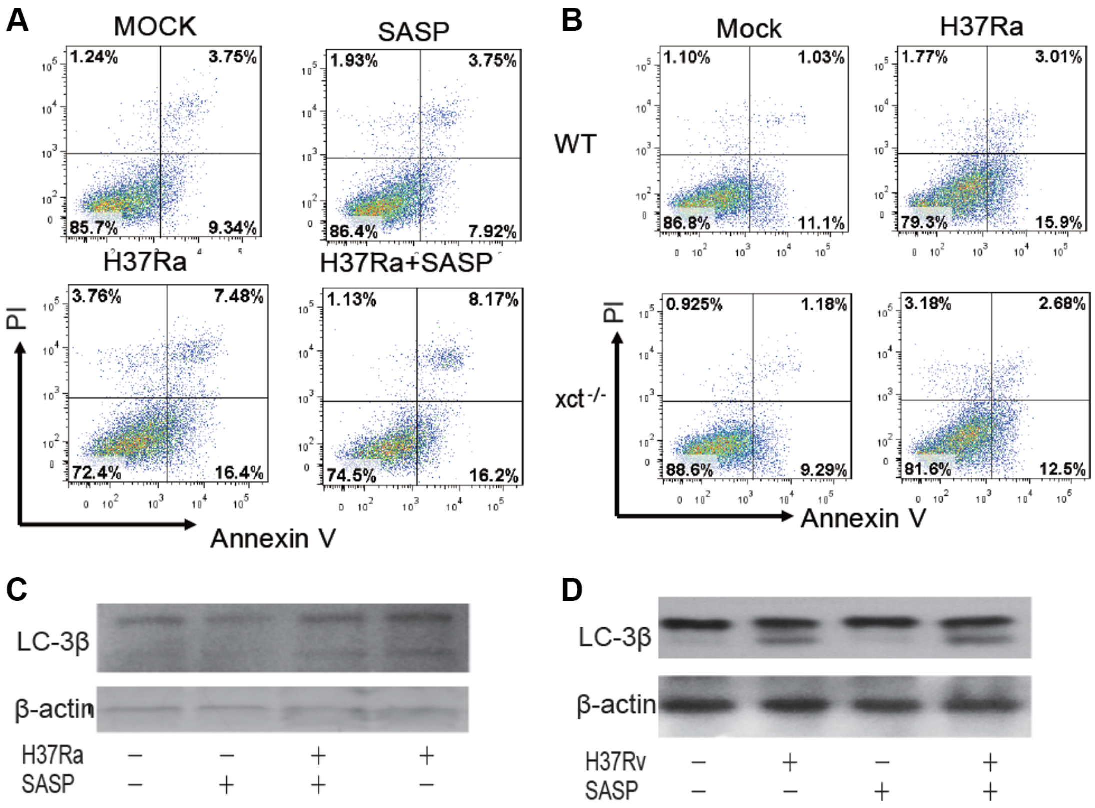

Supplementary Figure S4: Disruption of xCT did not affect apoptosis and autophagy of macrophage infected with Mtb. U937 macrophages pretreated with or without SASP (**A**) and peritoneal macrophages from WT and *xCT*$^{-/-}$ (**B**) were infected with H37Ra. After 24 h, cells apoptosis was analyzed by FACS. (**C–D**) U937 macrophages pretreated with or without SASP were and infected with H37Ra (C) or H37Rv (D). After 24 h, cells were harvest for immublotting of LC3β or β-acitn.

Supplementary Table S1: The demographic characteristics of the study populations

Study cohort	*n*	Age years[c]	M/F	Sputum Mtb culture (−/+)
TB[a]	3	45.0 (41.0−52.0)	1/2	0/3
TB	20	31.4 (20.0−56.0)	12/8	0/20
LTBI	20	29.6 (18.0−52.0)	11/9	ND[b]
HC	20	27.9 (18.0−46.0)	10/10	ND

[a]Lung biopsy specimens use for Immunohistochemistry.
[b]"ND" indicated not done.
[c]median/interquartile range.

Supplementary Table S2: Primer list

Gene	Primer (5′-3′)
mxCTf[a]	TTGGAGCCCTGTCCTATGC
mxCTr	CGAGCAGTTCCACCCAGAC
mcxcl5f	GGCATTTCTGTTGCTGTT
mcxcl5r	GCGATCAATTTGGGATTA
mcxcl1f	GGCTGGGATTCACCTCAA
mcxcl1r	GGCTATGACTTCGGTTTGG
mcxcl2f	ACCAACCACCAGGCTACA
mcxcl2r	CTTCAGGGTCAAGGCAAA
mcxcr2f	CCATCATCTATGCCTTTATT
mcxcr2r	GTTTGCTGAAGACGAGCT
mTNFaf	CCAGGCGGTGCTTGTTC
mTNFar	GGCTACAGGCTTGTCACTCG
mCCl2f	CATCTGCCCTAAGGTCTTC
mCCl2r	AGTGCTTGAGGTGGTTGTG
mIL-6f	CTTCTTGGGACTGATGCTG
mIL-6r	GGTCTGTTGGGAGTGGTAT
mIL-1bf	GAGCATCCAGCTTCAAATC
mIL-1br	GCTTCTCCACAGCCACAAT
mCCl5f	ACACCACTCCCTGCTGCTT
mCCl5r	GATGTATTCTTGAACCCACTTCTT
mIFNgf	AGCAACAACATAAGCGTCAT
mIFNgr	CCTCAAACTTGGCAATACTCA
mIL-10f	GACAACATACTGCTAACCGACTC
mIL-10R	TGGATCATTTCCGATAAGG
mIL12p40f	CCCCATTCCTACTTCTCCC
mIL12p40r	ACGCACCTTTCTGGTTACAC
mgapdhf	CAAATTCAACGGCACAGTCA
mgapdhr	TCTCGCTCCTGGAAGATGG
hcxcl5f[b]	TACAGACCACGCAAGGAGTT
hcxcl5r	TCTTCAGGGAGGCTACCAC
hGAPDHf	GACAGTCAGCCGCATCTTCT

hGAPDHr	TTAAAAGCAGCCCTGGTGAC
hcxcl1f	CCCCAAGAACATCCAAAGTG
hcxcl1r	GATGCAGGATTGAGGCAAG
hcxcl2f	CCAGTGCTTGCAGACCCT
hcxcl2r	GCATCTTTTCGATGATTTTCTT
hcxcr2f	AAACTCCCTCGTGATGCT
hcxcr2r	CACAGGAATGTGCCAAAA
hxCTf	CCATTGGCTATGTGCTGACA
hxCTr	CAGGAGAGGGCAACAAAGAT

[a]"m" stands for mouse; [b]"h" stands for human.
